# Novel use of *Quillaja saponaria* extracts to prevent viral infections in Atlantic salmon: *in vitro* ISAv and IPNv inhibition and *in vivo* protection against IPNv

**DOI:** 10.3389/fvets.2026.1688908

**Published:** 2026-01-29

**Authors:** Trinidad Schlotterbeck, Hernán Cortés, Hernan Cañon-Jones, Ricardo San Martín, Leandro Padilla, Mario Castillo-Ruiz

**Affiliations:** 1 Desert King, Valparaíso, Chile; 2 Faculty of Veterinary Medicine and Agronomy, Universidad de Las Americas, Santiago, Chile; 3 Sutardja Center for Entrepreneurship and Technology, College of Engineering, University of California, Berkeley, Berkeley, CA, United States; 4 Escuela de Tecnología Médica, Departamento de Ciencias Químicas y Biológicas, Facultad de Ciencias de la Salud, Universidad Bernardo O’Higgins, Santiago, Chile; 5 Escuela de Química y Farmacia, Facultad de Medicina, Universidad Andres Bello, Santiago, Chile

**Keywords:** antiviral, infectious diseases, IPNv, ISAv, Quillaja, saponin, sustainable aquaculture

## Abstract

**Introduction:**

The global expansion of salmon farming has increased the prevalence of viral diseases, especially Infectious Pancreatic Necrosis (IPN) and Infectious Salmon Anemia (ISA), posing significant threats to the sustainability and productivity of the industry. Extracts from the Chilean tree *Quillaja saponaria* Molina are a rich source of triterpenic saponins, glycosides with antimicrobial and immunomodulatory properties. This study assessed the antiviral efficacy of a non-purified (NPQ), a partially purified (PPQ, the active ingredient of PAQ-Xtract®), and a purified (VaxSap®) commercial *Quillaja saponaria* extracts, as dietary additives to prevent and control viral infections in Atlantic salmon (*Salmo salar*).

**Methods:**

The *In vitro* antiviral activity of the extracts was measured through infection assay in ASK and CHSE-214 cell lines. Results were expressed as the percentage of inhibition, comparing the number of viral copies or the number of PFU produced in untreated conditions to those in *Quillaja saponaria* extracts treated conditions. *In vivo* trial were conducted on Atlantic salmon fry. During the trial, fish were fed with a standard diet and supplemented with NPQ or PPQ. Fishes were intraperitoneal challenged with IPNv and mortalitiy registered.

**Results:**

*In vitro* assays demonstrated that *Quillaja* extracts generated over 90 and 60% inhibition of replication of ISAv and IPNv, respectively. *In vivo* challenge experiments further showed that dietary supplementation with NPQ or PPQ significantly reduced cumulative mortality following IPNv infection compared with untreated controls.

**Discussion:**

These findings indicate that *Quillaja saponaria* extracts may be used as effective, natural, non-pharmacological additives to prevent diseases caused by these viruses. Their use supports the development of sustainable and environmentally responsible disease-management strategies in modern aquaculture.

## 1 Introduction

Aquaculture has seen a significant expansion globally, possibly driven by the rising human population demanding fish protein sources and the plateau in fisheries (1). In Chile, salmon farming has become one of the country’s most important economic activities; Chile currently is the second producer of salmon worldwide after Norway (1, 2). As in other intensive animal production systems like poultry or pig production (3), the rapid expansion of salmon farming has also been accompanied by the emergence of infectious diseases, which pose significant threats to the stability and profitability of the industry (4). Various bacterial and viral pathogens have been identified as key contributors to decreasing production efficiency and increasing mortality rates in salmonids (5, 6). These infections not only disrupt the economic viability of salmon aquaculture but also lead to greater dependency on antibiotics and other treatments, intensifying environmental and health concerns (7). Viral infectious diseases such as Infectious pancreatic necrosis virus (IPNV) and Infectious salmon anemia virus (ISAv) are challenging in terms of growth capability of salmon farming industry in Chile (8).

IPN is a highly contagious disease that causes high mortality in fry during their first feeding stage and in juveniles following transfer to seawater (9). This disease is one of the most important fresh water viral diseases in Chile. Its etiological agent is the IPN virus (IPNv) belongs to the family *Birnaviridae*, genus *Aquabirnavirus*, currently classified by ICTV as the species *Aquabirnavirus salmonidae* and is characterized by non-enveloped capsid with a genome consisting of double stranded RNA. Clinical signs of the disease are acute, subacute or chronic and include swelling of the abdomen and eyes, darkening of the skin, necrosis of the pancreatic tissue and spiral swim and mortality (10, 11).

Another viral agent in the aquaculture industry is ISA virus (ISAv) affecting salmonids and leading to high mortalities, threatening the main areas of fish farming in the North Atlantic Ocean and in Chile. In 2007, the Chilean salmon farming industry was strongly impacted by the first outbreak of ISA causing 69% of biomass losses (12); this event was caused by a variant deletion in a genomic segment and warns about the ability of this virus to adapt, change, and be a threat to the industry (13). The ISAv belongs to the family *Orthomyxoviridae*, genus *Isavirus*, classified by ICTV as the species *Isavirus salaris.*, and has a single-stranded RNA genome with envelope (11, 14). Once the virus distributes inside the fish, it infects all internal organs, preferably the endothelium, causing clinical signs such as bleeding, lethargy, abdominal distension, and severe anemia. Mortality by this disease is high and few fish remain alive as carriers (12).

The main strategies currently used to prevent and control the diseases caused by virus in salmon producing facilities are listed as follows: strict control of biosafety conditions, selection of resistant fish specimens for further breeding, stocking practices, functional diets, use of vaccines and careful hygiene management (15–17). The selective breeding of salmon for resistance to IPNv using QTL marker, described as a genomic locus conferring resistance to IPNv infection in Atlantic salmon (17, 18). Genetically markers have highlighted how immunoresponse is a key factor for resistance/susceptibility of fish, as well as the existence of a high variability based on the interaction pathogen-host-environment, that will define the response to viral infections (19, 20).

Functional foods have been implemented for the improvement of fish health and their resistance to diseases (19, 20). Different diets containing microalgae rich in polyunsaturated fatty acids, glycans, carotenoids, among others have been tested (21). These ingredients can promote fish welfare while improving intestinal health and by increasing the resistance to diseases (21–24). Therefore, addressing viral infectious challenges through innovative solutions, such as functional diets designed to enhance fish immunity, is critical to ensuring the long-term sustainability and resilience of the salmon farming sector.

*Quillaja saponaria* (Molina) is a native tree of Chile, used as source of raw material to produce triterpenic saponins, a family of tensoactive glycosides having outstanding immunostimulating properties (25). Saponins can be obtained industrially as powder or liquid products, and may be in an unpurified, partially, or purified state, mainly used as natural emulsifiers in cosmetics, food, and beverages (25). *Quillaja saponaria* saponins have been extensively utilized in various applications due to their antibacterial, antiviral, antifungal, and antiparasitic properties (26–28). Different studies into their structure and mode of action have revealed that a significant aspect of their antimicrobial efficacy is derived from their interaction with biological membranes, particularly through binding cholesterol (25). This interaction disrupts the viral envelope, compromises membrane integrity (29) and inhibits the ability of viruses and bacteria to adhere to and complete the infective cycle (30). For example, triterpenoid saponins have been shown to inactivate influenza, herpes simplex, and coronaviruses by impairing the fusion step and reducing viral infectivity (6, 7). Moreover, experimental results show that saponins provide robust protection against viruses in both laboratory animals and cultured cell lines. Importantly, these antiviral effects are achieved at concentrations lower than those required to induce cytotoxicity, suggesting a favorable therapeutic window for saponins in antiviral applications (31).

Specifically, *Quillaja* saponins are amphipathic glycosides, characterized by a hydrophobic aglycone (triterpenoid or steroidal sapogenin) linked to hydrophilic sugar chains. This dual nature confers them the ability to interact with biological membranes, forming complexes with sterols and modulating cell signaling pathways. Beyond their well-known surfactant and adjuvant properties, extensive evidence supports that saponins exert broad-spectrum antiviral activities through both direct virucidal effects and host-mediated immune mechanisms (32). Additionally, saponins can affect post-entry events, such as viral genome replication and protein synthesis. Studies on triterpene-rich extracts have reported reductions in plaque size and progeny production even when added after viral adsorption, suggesting intracellular antiviral activity (18).

Based on the description above, the hypothesis of this study was that *Quillaja saponaria* extracts have the ability to reduce the infective capacity of IPNv and ISAv. Therefore, this work aimed to assess the *in vitro* and *in vivo* antiviral efficacy of commercial *Quillaja* saponin extracts in Atlantic salmon, focusing on their application as an oral supplement to prevent and control viral diseases in aquaculture. This approach seeks to contribute to the development of sustainable and eco-friendly strategies for disease management, offering alternative solutions that support responsible and environmentally conscious aquaculture production.

## 2 Materials and methods

### 2.1 Preparation and characterization of *Quillaja saponaria* products

All *Quillaja saponaria* products were obtained from Desert King Chile S. A., Chile. Three commercial powder products with different levels of purification of saponins were used in the *in vitro* trials: (a) VaxSap® (highly purified extract containing 90% w/w saponins); (b) Partially-purified *Quillaja saponaria* extract (PPQ) containing 65% saponins (which corresponds to the active ingredient of the commercially available product PAQ-Xtract), and (c) Non-purified *Quillaja saponaria* extract with 25% saponins (NPQ), which means 2.5 times lower level of purified saponins compared to PPQ. Saponin concentration was determined by RP-UPLC [31]. Briefly, an aliquot from the product was filtered (0.22 μm) and analyzed on a C18 column (particle size 1.7 μm, inner diameter 2.1 mm, length 50 mm) at 30° C. The samples were eluted with mobile phase flow-rate of 0.41 mL/min, using a liquid gradient consisting of 34–45% HPLC grade acetonitrile in deionized water with 0.15% v/v formic acid. The elution was monitored with an absorbance detector tuned to 210 nm. A highly purified *Quillaja saponaria* extract (containing 90% w/w saponins, supplied by Desert King Chile) was used as standard for saponin quantification.

### 2.2 *In vitro* antiviral activity of *Quillaja saponaria* extracts against ISAv

ISAv assays were performed in the ASK cell line, which is highly permissive and cytopathic for ISAv. Viral cytopathic effects (CPE) were monitored daily by light microscopy, and characteristic CPE were observed in virus-only controls. Uninfected controls, infected controls without treatment, and PCR no-template controls were included in all assays. Viral infection was carried out using 1 × 10^6^ cells ASK cell line (ATCC® CRL2747™) into 6-well plate to a cell confluence of 80 to 90%. The cells were cultivated at 16 °C in Leibovitz medium (L-15, Hyclone, Thermo Scientific), supplemented with gentamicin (50 μg/mL), L-glutamine (4 mM, Gibco, Thermo Scientific), 2-mercaptoethanol 1% (v/v, 2-ME, Gibco), fetal bovine serum 10% (v/v, FBS, Hyclone). Antiviral efficacy was determined by incubating the cells for 4 h with the viral inoculum (Chilean isolate a viral titer of 10^6^ copies of viral RNA) at MOI = 0.01 (33). The ISAv strain used was isolated from *Salmo salar* in southern Chile, genogroup HPR7b, characterized as virulent. The culture medium was subsequently removed and fresh culture medium supplemented with antibiotics and different *Quillaja saponaria* extracts, and as control, inoculum without *Quillaja saponaria* extracts treatment was used. Viral RNA from the supernatant of infected ASK cells was extracted using the EZNA Total RNA Kit I (Omega Bio-tek), and the viral titer (copies/mL) was quantified by qRT-PCR using F5/R5 primers following the procedure described by Rivas-Aravena et al. (33). All experiments were done in triplicate. Results were expressed as the percentage of inhibition, comparing the number of viral copies produced in untreated conditions to those in *Quillaja saponaria* extracts treated conditions. Cytotoxicity of the products was performed previously. Briefly, cytotoxicity was assessed in SHK-1 cells cultured in supplemented L-15 medium at 15 °C. After reaching confluence, cells were exposed to the *Quillaja saponaria* extracts for 24 h, washed, detached, and analyzed by flow cytometry using propidium iodide incorporation as a marker of nonviable cells. Ethanol-treated and untreated cells served as positive and negative controls, respectively, and CC₅₀ and CC₉₀ values were determined by probit analysis (31). Additionally, a concentration of 10 μg/mL *Quillaja saponaria* extracts were evaluated in ASK cell. Twenty-four hours after treatment, propidium iodine intake, RNA integrity and cell recovery were assesed. Total RNA was extracted following the TRIsure protocol (Bioline). Cell recovery and morphology were evaluated after 96 h using inverted light microscopy.

### 2.3 *In vitro* antiviral activity of *Quillaja saponaria* extracts against IPN

The antiviral activity of the extracts was measured through infection assay in CHSE-214 cell monolayers derived from salmon (*Oncorhynchus tshawytscha*, ATCC Number CRL-1681, American Type Culture Collection). Cell plates were incubated in 24 well plates until a 90% cell confluence, at 16 °C in MEM culture medium MEM (Gibco) supplemented with 10% Fetal Bovine Serum (Hyclone), 2 mM L-glutamine (Gibco), 10 mM HEPES (Hyclone), 100 IU mL-1/100 ug/mL-1 of gentamicin (Gibco). Antiviral activity was measured by first removing cell culture medium, and infecting cell with a viral suspension of a Chilean IPNv isolated at MOI = 0.05 (33), an approximate 50 plaque forming units (PFU) and adding the different *Quillaja saponaria* extracts, and as control, inoculum without *Quillaja saponaria* extracts treatment was used. The IPNv strain corresponds to a field isolated from *Salmo salar*, genogroup Sp (serotype A2). After 1 h of viral adsorption at 15 °C, the inoculum was removed, and the cell monolayer was covered with agarose gel of low temperature of gelation at 0.5% in growth medium supplemented with different *Quillaja saponaria* extracts. Plates were further incubated for 3 days at 15 °C and then the cells were fixed with 1 mL of 37% formaldehyde at room temperature for 1 h. After removing the fixative and the agarose overlay, the cell monolayer was stained with crystal violet solution 0.5% for 1 h. Finally, cells were washed with water and plaque forming units (PFU) were counted (34). Each condition was performed in triplicate. Results were expressed as the percentage of inhibition, comparing the number of PFU produced in untreated conditions to those in *Quillaja saponaria* extracts treated conditions.

### 2.4 *In vivo* efficacy of orally given *Quillaja saponaria* extracts against an IPNv intraperitoneal challenge in freshwater conditions

**Figure 1:**
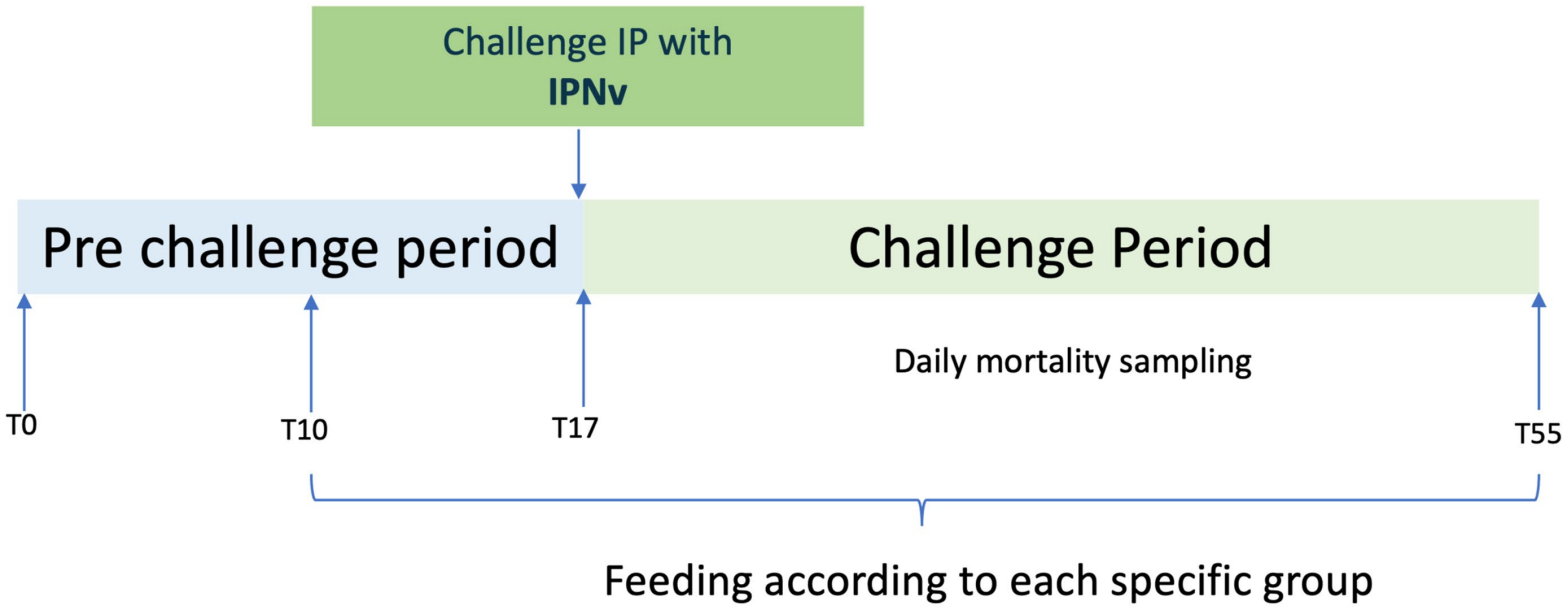
Schematic representation of the experimental timeline, including acclimation period, group-specific feeding, challenge, and subsequent monitoring period.

*In vivo* trials were conducted at ACTIVAQ laboratory in Universidad de Santiago de Chile, Chile. Two hundred and forty (240) Atlantic salmon fry (5.5 ± 0.4 g/each) were used. All fish were clinically healthy and no IPNv or *Piscirickettsiasalmonis* (intracellular pathogen that causes piscirickettsiosis) were detected by RT-PCR. Sixty (60) fish were sampled to check gill, intestine and skin. Sampling for bacterial kidney disease (BKD) and *Piscirickettsia salmonis* was carried out using Gram staining (spleen, kidney, and brain), acridine orange (gills) and IFAT analysis (35, 36). RT-PCR was used for the detection of IPNv (37). During the trial, fish were fed with a diet of 15 micro EWOS 15CP® (50.0% total crude protein, 22% lipids, 1.0% crude fiber, 9.0% moisture, 9.5% ash and 8.5% nitrogen-free extract), at 0.75% of body weight/day (bw/day). For these *in vivo* trials, NPQ and PPQ were used. These were included in the diet at a dose of 3,75 mg of product per kg body weight/day, which represents 0.94 mg saponins/kg body weight/day for NPQ and 2.4 mg saponins/kg body weight/day of PPQ. The following experimental groups were used: (a) negative control (fish not challenged with virus and not fed with *Quillaja saponaria* products); (b) positive control (fish challenged with the virus and fed without *Quillaja saponaria* products); (c) NPQ group and not challenged with virus; (d) NPQ group and challenged with virus; (e) PPQ group and not challenged with the virus, (f) PPQ group and challenged with the virus. All groups were tested in duplicate, consisting of two independent replicate tanks per condition. Sample size was defined based on expected differences in cumulative mortality from previous IPNv challenges, resulting in 20 fish per group. Each treatment was maintained in an independent tank to avoid pseudoreplication. Fish were randomly assigned to tanks and dietary treatments, and personnel responsible for mortality monitoring and virological analyses were blinded to treatment allocation. The challenge was conducted by intraperitoneal injection with a standardized viral dose to ensure synchronized and homogeneous infection across individuals. The challenge was via an intra-peritoneal injection of 0.1 mL of inoculum of IPNv (1 × 10^8^ viral genome copies/mL) in the ventral line in each fish. Negative control fish were inoculated with 0.1 mL of culture medium. A feeding ten (10) day acclimation period using only commercial feeding and no *Quillaja saponaria* products was carried out, followed by 45 days of treatment with orally administered *Quillaja saponaria* products. An infection with an IPNv was carried out on day 7 post-acclimation. Mortality was registered daily, and total mortality was calculated at the end of the trial (Figure 1).

### 2.5 Statistical analysis

*In vitro* antiviral data were analyzed using continuous outcomes. Percentage inhibition values were compared among treatments using one-way analysis of variance (ANOVA), followed by Tukey’s post-hoc test. Data are presented as mean ± standard deviation (SD). Different superscript letters indicate statistically significant differences between groups at *p* < 0.05. *In vivo* mortality data were analyzed using a generalized linear mixed model (GLMM). Cumulative mortality was analyzed using two complementary approaches addressing both absolute mortality outcomes and relative mortality risk. First, mortality risk was assessed using a binomial generalized linear mixed model (GLMM) with a logit link function, where individual fish status (dead/alive) was used as the response variable. Dietary treatment was included as a fixed effect, and tank was included as a random effect to account for experimental clustering. Model-estimated marginal means were obtained on the response scale and are presented as cumulative mortality percentages, providing an estimate of the absolute magnitude of mortality under each treatment. In addition, treatment effects were quantified in terms of relative mortality risk using odds ratios (OR). Odds ratios and their corresponding 95% confidence intervals were calculated by back-transformation from the log-odds scale of the GLMM fixed-effect coefficients and are visualized as a forest plot, where OR values below 1 indicate reduced odds of mortality relative to the infected untreated control (PC_IPNv). Hypothesis-driven *post hoc* comparisons were performed using Dunnett’s adjustment to compare Quillaja-supplemented diets under IPNv challenge (NPQ_IPNv and PPQ_IPNv) against the infected untreated control (PC_IPNv). Finally, exploratory pairwise comparisons among all treatment groups were conducted using Tukey-adjusted *post hoc* tests. All analyses were conducted in R. version 4.5.2.

## 3 Results

### 3.1 *In vitro* efficacy of *Quillaja saponaria* extracts against IPNv and ISAv infections

*Quillaja saponaria* extracts could effectively reduce the virus proliferation and infection in monolayers of ISAv and IPNv (Tables 1, 2). For ISAv, previous proof-of-concept assays were performed using 0.24, 0.12, 0.06, and 0.03 μg/mL. Lower concentrations (0.06 and 0.12 μg/mL) showed no inhibitory effect, and at 0.24 μg/mL, inhibition levels ranged from 5% with VaxSap® to 48% with NPQ. Given these findings and with a target inhibition threshold of at least 50%, the later assays were conducted at a concentration of 0.48 μg/mL for the different products. At that concentration, inhibition rates between 95 and 99% were found for all products tested. All *Quillaja saponaria* extracts tested at 10 μg/mL preserved RNA integrity and allowed normal recovery and propagation of ASK cells after replacement with fresh medium (data not shown). These results indicate that *Quillaja saponaria* extracts do not induce cytotoxic effects under the conditions tested, and that the observed antiviral activity is not attributable to nonspecific cellular toxicity.

**Table 1:** *In vitro* efficacy of different *Quillaja saponaria* extracts against ISAv infections in ASK cells.

Product	Product concentration (μg/mL)	Saponin concentration (μg/mL)	Viral copies/mL (10^4^)	Infection inhibition (%)
Control	0	0	870.818+/−15.903	0^a^
VaxSap®	0.48	0.43	0.0005+/−0.0003	>99^b^
PPQ	0.48	0.31	44.800+/−24.335	95^b^
NPQ	0.48	0.12	0.010+/−0.007	>99^b^

Data are presented as mean ± SD. Statistical analysis was performed using one-way ANOVA followed by Tukey’s post-hoc test. Different superscript letters (a, b) indicate statistically significant differences between groups at *p* < 0.05.

**Table 2:** *In vitro* efficacy of different *Quillaja saponaria* extracts against IPNv infections in CHSE-214 cells.

Product	Product concentration (μg/mL)	Saponin concentration (μg/mL)	Infection inhibition (%)
Control	0	0	0^c^
VaxSap®	0.12	0.1	75+/−3.9^a^
PPQ	0.24	0.15	69.5+/−1.3^ab^
NPQ	0.24	0.073	59.1+/−1.2^b^

Data are presented as mean ± SD. Statistical analysis was performed using one-way ANOVA followed by Tukey’s post-hoc test. Groups sharing the same superscript letter are not significantly different, whereas different letters (a, b, c) indicate statistically significant differences at *p* < 0.05.

Regarding IPNv, VaxSap®, PPQ, and NPQ showed high inhibition efficacy at doses of 0.12, 0.24, and 0.24 μg/mL, respectively (Table 2). *Quillaja saponaria* extracts effectively controlled *in vitro* infection, significantly reducing PFU compared to untreated control. In addition, to determine whether the antiviral activity of *Quillaja saponaria* extracts was independent of cytotoxicity, we calculated selectivity index (SI = CC₅₀ / EC₅₀). Since the referenced IPNv efficacy dataset (31) provides only single antiviral concentrations, we conservatively estimated SI using the concentrations that produced >50% infection inhibition (C_eff). Using our CC₅₀ values (20 μg/mL for VaxSap®, 22 μg/mL for PPQ UD100Q, the active ingredient of PAQ-Xtract®), and 85 μg/mL for NPQ (QD100), and the corresponding C_eff values (0.12–0.24 μg/mL), we obtained SI_min values of 166.7 for VaxSap®,91.7 for UD100Q, and 354.2 for QD100 (Supplementary Table 1). Because these doses already produced 59–75% inhibition, the true EC₅₀ values must be lower than C_eff; consequently, the real SI values are likely even higher. These data demonstrate a wide therapeutic window and confirm that antiviral activity is achieved at concentrations far below those associated with cytotoxicity.

### 3.2 *In vivo* efficacy of orally given *Quillaja saponaria* extracts against an IPNv intraperitoneal challenge

**Figure 2:**
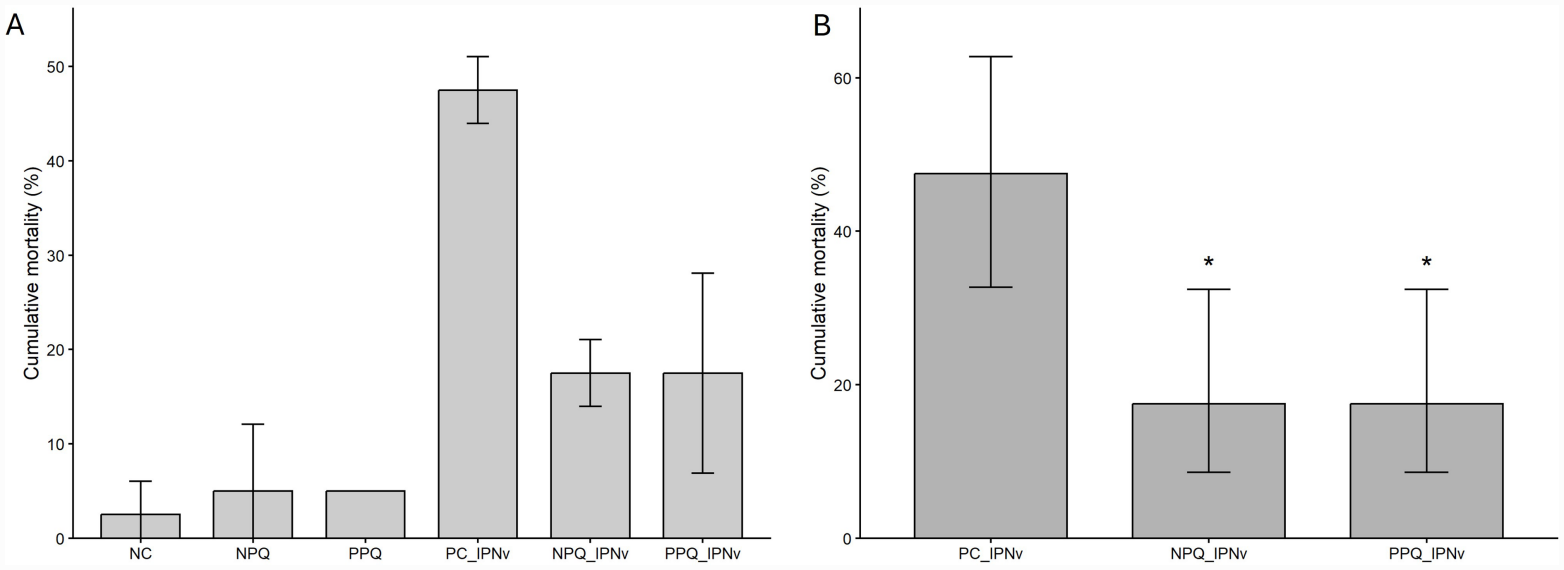
Mortality (%) in Atlantic salmon supplemented with *Quillaja saponaria* extracts NPQ and PPQ following IPNv challenge. **(A)** Descriptive cumulative mortality across dietary treatments with and without IPNv challenge. Bars represent mean cumulative mortality (%) per tank, with error bars indicating standard deviation. **(B)** Cumulative mortality following IPNv challenge under different dietary treatments. Bars represent model-estimated cumulative mortality (%) derived from a binomial generalized linear mixed model (GLMM) with tank as a random effect. Error bars indicate 95% confidence intervals. Asterisks denote statistically significant differences relative to the infected untreated control (PC_IPNv), based on Dunnett-adjusted *post hoc* comparisons (*p* < 0.05).

In the *in vivo* trials were performed according to the schematic diagram shown in Figure 1. Fish receiving either the NPQ or PPQ diets displayed lower mortality under IPNv challenge compared with the control group without *Quillaja saponaria* extracts treatment (Figure 2). There were no deaths during the 10-day acclimation period. Throughout the treatment period, the fish adequately consumed the food. The fish showed a similar weight at the end of the trial (control 6.94 g; NPQ 6.98; PPQ 7.72 g on average). In addition, there was no mortality associated with IPNv inoculation (day 17). Mortality began in the infected and untreated aquariums on day 33 and the outbreak lasted 3 days. Overall, deaths were recorded between days 33 and 41 of the trial. All sick fish exhibited nonspecific clinical signs. Ead fish were necropsied, revealing signs such as pale liver and necrotic spleen. All dead fish that had been challenged tested positive for IPNv by RT-PCR. Additionally, the infection was confirmed by monolayer infection of CHSE-214 from a macerate of organs from infected and dead fish.

**Figure 3:**
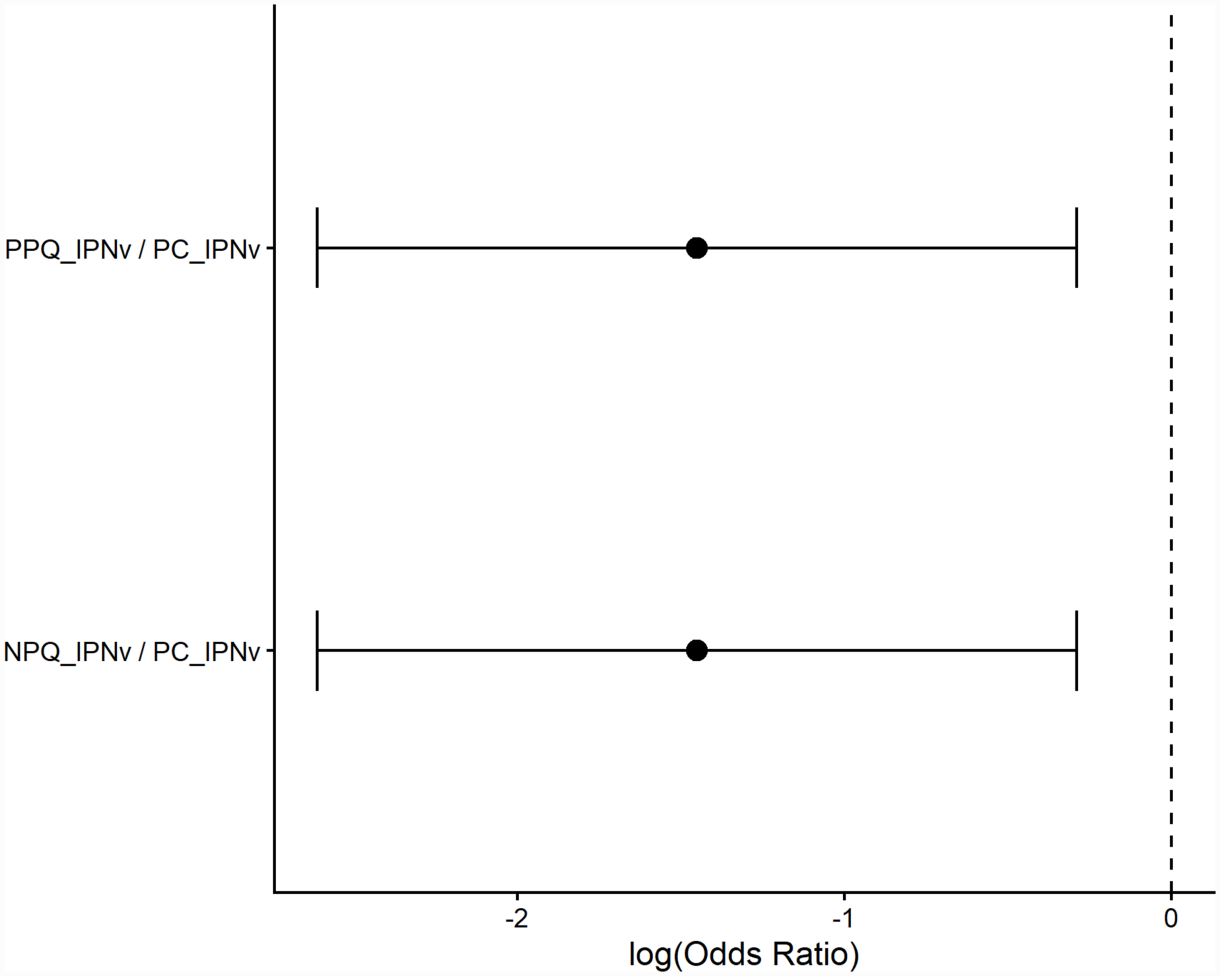
Effect of *Quillaja saponaria* extracts supplementation on mortality risk following IPNv challenge. Forest plot showing odds ratios (OR) and 95% confidence intervals derived from a binomial generalized linear mixed model (GLMM) with dietary treatment as a fixed effect and tank as a random effect. Odds ratios are expressed relative to the infected untreated control (PC_IPNv) using Dunnett-adjusted post hoc comparisons. Values of OR < 1 indicate reduced odds of mortality compared with PC_IPNv. Points represent model-estimated effects on the log-odds scale, with horizontal lines indicating 95% confidence intervals. Exact Dunnett-adjusted *p*-values are reported in Supplementary Table 3.

Descriptive analysis of cumulative mortality across all dietary treatments, including non-challenged controls, is shown in Figure 2A and Supplementary Table 2. Final cumulative mortality differed markedly among treatments. PC_IPNv (infected control) showed the highest mortality (45–50% per tank), dietary supplementation with Quillaja extracts reduced mortality following IPNv challenge: PPQ_IPNv tanks exhibited 10–25% mortality and NPQ_IPNv tanks 15–20%. GLMM-based inferential analysis demonstrated that dietary supplementation with *Quillaja saponaria* extracts significantly reduced cumulative mortality following IPNv challenge while accounting for tank-to-tank variability (Figure 2B). Beyond absolute mortality estimates, odds ratio analysis further quantified the protective effect of *Quillaja saponaria* extracts supplementation under IPNv challenge. Using the same binomial GLMM framework with tank included as a random intercept, both NPQ_IPNv and PPQ_IPNv treatments were associated with a pronounced reduction in mortality risk relative to PC_IPNv (OR = 0.234; 95% CI: 0.073–0.749; Dunnett-adjusted *p* = 0.0108 for both contrasts), corresponding to an approximate 75–80% reduction in the odds of mortality (Figure 3; Supplementary Table 3). In addition, exploratory Tukey-adjusted pairwise comparisons among all treatment groups showed in Supplementary Table 4.

## 4 Discussion

The present study confirms that *Quillaja saponaria* extracts have antiviral properties against ISAv and IPNv. *In vitro* trials using VaxSap®, PPQ, and NPQ demonstrated significant antiviral activity, preventing over 59% of IPNv infections and over 90% of ISAv infections at concentrations below 1.0 μg/mL in cell cultures. These effective doses were remarkably lower by a factor of 43 to 348 than the cytotoxic concentrations previously reported (31).

*In vitro* trials showed that *Quillaja saponaria* extracts were more effective at reducing infection in the enveloped ISAv than the non-enveloped IPNv, though both virus types were susceptible to all products. For ISAv, at 0.48 mg/mL, the three products demonstrated similar efficacy, suggesting that saponin fraction alone does not explain the results, as products with higher saponin concentrations did not yield greater inhibition, or that a minimum saponin concentration is required to achieve the inhibition of viruses. Although infectivity titration was not performed, the ISAv strain produced clear CPE in ASK cells, confirming productive infection under the experimental conditions. Future studies could incorporate this analysis to differentiate between infectious particles a non-infectious particles. For IPNv, at 0.24 mg/mL, reductions ranged from 59.1% (NPQ) to 75% (VaxSap®), indicating that more purified, saponin-rich extracts enhance better protection. These results are in agreement to previous *in vitro* studies using *Quillaja* saponins against other non-enveloped viruses as Reovirus (RV), Rhesus Rotavirus (RRV), and enveloped ones as Vaccinia (VV), Varicella-Zoster (VZV), Herpes Simplex Virus type 1 (HSV-1), Human Immunodeficiency Virus 1 & 2 (HIV-1, HIV-2) in different cell lines (29, 38). Later studies also found that the non-enveloped viruses were more resistant to the *Quillaja saponaria* extracts, and that all enveloped viruses were more susceptible, at doses under 1.0 mg/mL. Troian et al., also demonstrated the antiviral activity of a purified *Quillaja saponaria* extract, Quil-A, and purified saponin fractions from *Quillaja* brasiliensis, on two single-stranded RNA enveloped viruses, the Yellow Fever Virus (YFV) and the Chikungunya Virus (CHIKV) from the Flavi. Using Vero cells, they found at 24 h, a cytotoxicity (CC50) between 18.1 and 25 μg/mL for Quil-A and FB respectively, while at 5.0 μg/mL they found an inhibition of infection of 75.26 and 100% for IFV and CHIKV with Quil-A, and 36.8 and 93% with FB for IFV and CHIKV (39). The results indicate that *Quillaja* extracts effectively reduce viral infection at doses below their cytotoxic threshold, with the strongest antiviral effects observed against the CHKV virus (39). The *Quillaja saponaria* extracts evaluated in this study exhibited high selectivity indices (SI_min = 91.7–354.2), combining our CC₅₀ data with the antiviral efficacy values reported in CHSE-214 cells. Although only single antiviral doses were available, these concentrations produced 59–75% inhibition, indicating that EC₅₀ values are even lower. Therefore, the true SI values for VaxSap®, PPQ and NPQ are expected to exceed the estimates reported here. NPQ showed the highest SI_min (≈354), consistent with its lower cytotoxicity and strong antiviral effect. These results suggest that inhibition of IPNv replication is not driven by nonspecific membrane damage but rather by specific virucidal or entry-blocking mechanisms consistent with known saponin–cholesterol and saponin–viral envelope interactions. Overall, the SI data reinforce that Quillaja extracts exert genuine antiviral activity at non-cytotoxic concentrations.

Regarding the *in vivo* trials, the findings of this research indicate that the diet supplementation with either NPQ or PPQ, significantly reduced the mortalities, presenting a 63% lower mortality with respect to the control group challenged with the IPNv (47.5% for the control vs. 17.5% for the groups with *Quillaja saponaria* products). Nonetheless, the IPNv challenge was conducted using an intraperitoneal injection model, rather than by immersion or cohabitation, which represent more natural infection routes, which may limit the extrapolation of these results to field conditions. Although the intraperitoneal model offers high reproducibility, synchronized exposure, and reduced inter-individual variability, it does not fully replicate natural transmission dynamics. Therefore, future studies will incorporate immersion and cohabitation challenges to validate the protective effects of Quillaja extracts under more realistic epidemiological conditions. However, the observed reductions in mortality odds in Quillaja-treated groups are biologically meaningful and consistent with the antiviral activity observed *in vitro* against IPNv. The concordance between *in vitro* inhibition of viral replication and reduced *in vivo* mortality supports the hypothesis that Quillaja-based formulations enhance host resistance to viral infection. In addition, *in vivo* antiviral activity of purified *Quillaja saponaria* extracts have been reported in other animal models, as against rhesus rotavirus (RRV) using a mouse model (29). That study established that at an oral dosage of 15 mg/mouse of saponin extract, RRV-induced diarrhea can be significantly reduced from 79 to 11% when mice are exposed to 500 PFU for five consecutive days. Additionally, while a reduction of RRV induced diarrhea depended both on the concentration of virus introduced and on the number of *Quillaja* saponin product given to each mouse, the severity and interval of diarrhea under a variety of conditions tested, in all the treated mice were greatly reduced when compared to those that did not receive the *Quillaja saponaria* product. Tam and Roner provide *in vitro* and *in vivo* evidence supporting the ability of *Quillaja* extracts to reduce viral infections (29). Their findings could indicate mechanistic evidence that *Quillaja* saponins can inhibit viral infection by blocking virus-host attachment, likely through disruption of cellular membrane proteins and/or viral receptors. *Quillaja* saponins may interfere with this process by modulating lipid membranes, as they form complexes with cholesterol, impacting membrane structure and fluidity (40, 41). Cholesterol’s role in viral infectivity is well-documented, including viruses like the enveloped influenza A, morphologically similar to ISAv, and non-enveloped rotavirus, like IPNv, where its depletion disrupts lipid domains and membrane integrity (42). Such mechanisms may explain the observed inhibition of IPNv and ISAv infections by *Quillaja* saponins, highlighting their potential antiviral application. Possible explanations behind the *in vitro* and *in vivo* results, could consider mechanisms of interference in the host-pathogen interaction at the cell membrane level, however additional research is necessary to determine a possible mechanism of action against these viruses, such as viral pretreatment, cell pretreatment, or time-of-addition assays, approaches to determine the specific stage of the viral cycle targeted by antiviral compounds. While the high selectivity indices observed indicate that the antiviral activity of *Quillaja saponaria* extracts occurs at non-cytotoxic concentrations, our data does not distinguish whether inhibition results from direct virucidal effects, interference with viral entry, or modulation of early replication steps. In addition, our previous studies showed that NPQ and PPQ, when delivered orally, were able to modulate both innate and adaptive immune markers before pathogen exposure. In two independent trials against *P. salmonis*, dietary supplementation with NPQ and PPQ significantly increased the expression of IFN-I, IFN-II, C3, and CD8 T-cell markers in head kidney and spleen prior to infection. Notably, both extracts upregulated IFN-I and CD8 expressions at 30 days under a cohabitation challenge model, indicating a capacity to prime antiviral and cytotoxic immune pathways (31, 43, 44). Future IPNv studies incorporating tissue-level viral load, histopathology, and gene expression (e.g., IFN-I, Mx, ISG15, CD8) will be essential to clarify whether similar immunomodulatory mechanisms occur in the antiviral context (45). Plant-derived natural compounds such as, alkaloids, flavonoids, saponins, and essential oils, have demonstrated efficacy against various viral pathogens in aquaculture (46, 47). These compounds may exert direct antiviral effects by interfering with multiple stages of the viral replication cycle, adsorption, endocytosis, intracellular transport, genome replication, protein synthesis and assembly, and viral release (21, 47, 48). In addition, by enhancing the host immune response, both innate and adaptive, this dual mechanism of action positions these compounds as strong candidates for improving fish health (49). Filifolinyl senecionate, a natural compound isolated from the resinous exudates of the plant *Heliotropium filifolium*, showed anti IPNv activity with EC50 160 μg/mL and a CC50 up to 400 μg/mL (50). Mycophenolic acid, isolated from *Penicillium stoloniferum* cultures as a fermentation product, was effective in preventing viral protein synthesis, inhibiting the IPNv replication (51). On the other hand, alpinone, a compound extracted from the *Heliotropium huascoense* plant, showed antiviral activity against ISAv. Valenzuela et al., showed that fishes inoculated with alpinone increased the transcriptional expression of TNF-alpha, IL-1, IFN-alpha, IFN-y, and TGF-beta1 (52). Another natural compound with demonstrated antiviral activity is isoliquiritigenin, a flavonoid from *Glycyrrhiza glabra. In vitro* results showed that isoliquiritigenin inhibits spring viremia of carp virus (SVCV) replication at non-cytotoxic concentrations, reducing viral load and cytopathic effects in EPC cells. In addition, *in vivo*, juvenile carp treated with isoliquiritigenin showed significantly lower viral loads and mortality rates following SVCV infection (47). All these findings highlight the potential of natural compounds and the potential of *Quillaja saponaria* extracts as promising candidates in the development of sustainable antiviral strategies for aquaculture. The demonstrated efficacy in both *in vitro* and *in vivo* models supports their use as preventive tools to control and reduce disease burden in salmon farming. Considering increasing environmental pressures, such as climate change, which alter host-pathogen-environment dynamics and contribute to the emergence and spread of infectious diseases, it becomes critical to integrate environmentally responsible approaches into disease management. Although the present study focused primarily on antiviral activity and survival outcomes, no behavioral alterations, feeding impairments, or external lesions were observed in any *Quillaja saponaria* extracts treatment group. Growth performance also remained comparable among treatments during the experimental period. Furthermore, *Quillaja saponaria* extracts of similar composition have been extensively evaluated in Atlantic salmon and other aquaculture species, consistently demonstrating an absence of toxicity at dietary inclusion levels below 150 mg/kg (44). Nevertheless, we acknowledge that the present study did not include formal histopathology or long-term growth evaluations, which future studies should incorporate.

The use of plant-derived compounds like *Quillaja* saponins aligns with eco-friendly production practices and supports the development of preventive strategies that enhance fish immune response and reduce the need for conventional pharmaceuticals. Ultimately, this contributes to more resilient and sustainable aquaculture systems, safeguarding animal health and welfare while reinforcing global food security. Further research could complement the previous findings to elucidate the mechanisms of action of *Quillaja saponaria* extracts, to support the salmon producer in the prevention and control of the different viral, bacteria, fungi and parasites challenges.

## 5 Conclusion

The present study evaluated the antiviral potential of different *Quillaja saponaria* extracts with different purification against two RNA viruses of major relevance to salmon aquaculture: ISAv and IPNv. The *in vitro assays* showed that all *Quillaja saponaria* extracts exhibited antiviral activity, particularly against ISAv, indicating that these extracts effectively interfere with viral replication. *In vivo* trials confirmed that diets supplemented with either PPQ (the active ingredient of PAQ-Xtract®) or NPQ products reduced mortality in the model of intra-peritoneal IPNv-challenged salmon. These findings suggest that *Quillaja saponaria* products could be a non-pharmacological alternative for improving the survival of fish to IPNv. Moreover, the use of these plant-derived compounds could represent a sustainable strategy that aligns with environmentally responsible aquaculture practices, contributing to the reduction of pharmaceutical dependence and supporting eco-friendly disease management approaches.

## Data Availability

The original contributions presented in the study are included in the article/Supplementary material, further inquiries can be directed to the corresponding author/s.
