# Supplementary material for: Novel use of Quillaja saponaria extracts to prevent viral infections in Atlantic salmon: in vitro ISAv and IPNv inhibition and in vivo protection against IPNv
Source: Front Vet Sci. 2026 Jan 29;13:1688908. doi: 10.3389/fvets.2026.1688908 (PMC12894021; doi:10.3389/fvets.2026.1688908)
Supplement: Supplementary file 1 [file Table_1.DOCX]

Supplementary table

**Table 1.** Selectivity Index (SI) Analysis for *Quillaja saponaria* extracts

| **Product (Extract)** | **CC₅₀ (µg/mL)*** | **C_eff for IPNv inhibition (µg/mL)**** | **Infection inhibition (%)** | **SI_min = CC₅₀ / C_eff** |
| --- | --- | --- | --- | --- |
| VaxSap | 20 | 0.12 | 75 ± 3.9 | 166.7 |
| PPQ | 22 | 0.24 | 69.5 ± 1.3 | 91.7 |
| NPQ | 85 | 0.24 | 59.1 ± 1.2 | 354.2 |

Conservative selectivity indices (SI = CC₅₀ / C_eff) of three *Quillaja saponaria* extracts against IPNv in CHSE-214 cells. Because only single antiviral concentrations were reported, C_eff values were used as upper-bound EC₅₀ estimates; therefore, SI_min values represent conservative lower-bound estimates of true SI.

* CC₅₀ values derive from our cytotoxicity assays in SHK-1, CHSE-214, MA104, and ASK cells.
 ** Single product concentrations yielding >50% inhibition of IPNv in CHSE-214 cells (Table 2 of Cañon et al., 2020).

**Table 2.** Efficacy of treatment with *Quillaja saponaria* extracts. Results showed the percentage of mortality in each aquarium.

| **Treatment** | **Initial Fishes** | **Final Fishes** | **% Mortality** |
| --- | --- | --- | --- |
| Negative Control (Normal Diet) | 20 | 19 | 5 |
| Negative Control (Normal Diet) | 20 | 0 | 0 |
| Positive Control (Normal Diet-IPNv challenged) | 20 | 10 | 50 |
| Positive Control (Normal Diet-IPNv challenged) | 20 | 11 | 45 |
| NPQ diet | 20 | 20 | 0 |
| NPQ diet | 20 | 18 | 10 |
| NPQ diet-IPNv challenged | 20 | 17 | 15 |
| NPQ diet-IPNv challenged | 20 | 16 | 20 |
| PPQ diet | 20 | 19 | 5 |
| PPQ diet | 20 | 19 | 5 |
| PPQ diet-IPNv challenged | 20 | 15 | 25 |
| PPQ diet-IPNv challenged | 20 | 18 | 10 |

**Table 3.** GLMM pairwise post hoc comparisons for *in vivo* mortality following IPNv challenge**.**

| **Comparison** | **Odds Ratio** | **95% CI (lower–upper)** | **z-ratio** | **p-value** |
| --- | --- | --- | --- | --- |
| NPQ_IPNv vs PC_IPNv | 0.234 | 0.073 – 0.749 | −2.77 | 0.0108 |
| PPQ_IPNv vs PC_IPNv | 0.234 | 0.073 – 0.749 | −2.77 | 0.0108 |

**Table 4.** Exploratory pairwise comparisons of mortality risk among all treatment groups (Tukey-adjusted)

| **Comparison** | **Odds Ratio (OR)** | **95% CI** | **Tukey-adjusted p-value** |
| --- | --- | --- | --- |
| PC_IPNv vs NC | 35.29 | 3.02 – 412.6 | 0.0102 |
| PC_IPNv vs NPQ | 17.19 | 2.14 – 138.2 | 0.0044 |
| PC_IPNv vs NPQ_IPNv | 4.27 | 0.94 – 19.32 | 0.0617 |
| PC_IPNv vs PPQ | 17.19 | 2.14 – 138.2 | 0.0044 |
| PC_IPNv vs PPQ_IPNv | 4.27 | 0.94 – 19.32 | 0.0617 |
| NC vs NPQ | 0.49 | 0.05 – 4.60 | 0.9925 |
| NC vs NPQ_IPNv | 0.12 | 0.02 – 0.91 | 0.3837 |
| NC vs PPQ | 0.49 | 0.05 – 4.60 | 0.9925 |
| NC vs PPQ_IPNv | 0.12 | 0.02 – 0.91 | 0.3837 |
| NPQ vs NPQ_IPNv | 0.25 | 0.04 – 1.40 | 0.5541 |
| NPQ vs PPQ | 1.00 | 0.15 – 6.62 | 1.000 |
| NPQ vs PPQ_IPNv | 0.25 | 0.04 – 1.40 | 0.5541 |
| NPQ_IPNv vs PPQ | 4.03 | 0.72 – 22.55 | 0.5540 |
| NPQ_IPNv vs PPQ_IPNv | 1.00 | 0.22 – 4.58 | 1.000 |
| PPQ vs PPQ_IPNv | 0.25 | 0.04 – 1.40 | 0.5540 |
